# Primary Biliary Cholangitis and CREST Syndrome: A Rare, Overlapping Presentation With a Review of the Literature

**DOI:** 10.7759/cureus.11986

**Published:** 2020-12-08

**Authors:** Shayan Iqbal Khan, Parkash Bachani, Amna Saleem, Arifa Jameel, Uzzam Ahmed Khawaja

**Affiliations:** 1 Medicine, Liaquat University of Medical and Health Sciences, Hyderabad, PAK; 2 Medicine, Jinnah Medical and Dental College, Karachi, PAK; 3 Internal Medicine, Liaquat University of Medical and Health Sciences, Hyderabad, PAK; 4 Internal Medicine, Jinnah Medical and Dental College, Karachi, PAK; 5 Clinical and Translational Research, Larkin Community Hospital, South Miami, USA

**Keywords:** primary biliary cholangitis, hepatitis c virus infection, extrahepatic manifestations, crest syndrome, limited systemic sclerosis

## Abstract

CREST (calcinosis, Raynaud's phenomenon, esophageal dysmotility, sclerodactyly, and telangiectasia) syndrome, also known as the limited cutaneous form of systemic sclerosis (lcSSc), is a multisystem connective tissue disorder often manifesting as a consequence of superimposed autoimmune hepatitis. Herein, we present a case of a 40-year-old female with a past one-year history of hepatitis C presenting with the chief complaints of progressive thickness and tightness of the skin of hands and face and dysphagia for the past three months, along with arthralgia of the hands for the past two months, suggestive of CREST syndrome. Through this case, we intend to emphasize the association between extrahepatic manifestations and the emergence of autoantibodies in patients with hepatitis C virus (HCV) infection and discuss the clinical relevance of the autoantibodies in extrahepatic disorders, in our case, CREST syndrome. It is well-known that chronic HCV infection plays a significant part in the production of non-organ-specific autoantibodies, including antinuclear antibodies (ANA) and smooth muscle antibodies, and organ-specific autoantibodies. Clinicians must be aware of the possibility of such liver damage in patients with systemic sclerosis.

## Introduction

Systemic sclerosis (SSc) is an unusual autoimmune, fibrotic, complex polygenetic connective tissue disease with an obscure origin, affecting the skin in all areas along with internal organs [1]. Literature suggests that activation of the immune system, accumulation of collagen, and vascular injury contributes to the progression of the pathology [2]. Based on the clinical and serological criteria there are two patterns of this disease, diffuse cutaneous sclerosis which involves the skin along with internal organs, and limited cutaneous sclerosis formerly known as CREST (calcinosis, Raynauds phenomena, esophageal dysmotility, sclerodactyly, and telangiectasia) syndrome [3].

While Raynaud's phenomenon, occurring as a result of vasoconstriction in response to mild cold and stress, is the initial finding of this disease, the evident physical manifestation is the thickening of the skin with a variety of patterns indicating the definitive diagnosis [4]. Limited cutaneous systemic sclerosis may involve the skin distal to the elbow mainly the fingers (sclerodactyly), distal knee, and face but spares the trunk, upper arm, and upper leg [5].

For diagnosis, anti-nuclear antibodies remain an unspecific antibody existing in 90% of the cases of systemic sclerosis, other antibodies such as anti-centromere, anti-SCL70, and anti-RNA polymerase III are more discrete [5]. Additionally, localized cutaneous systemic sclerosis shares an immune profile with primary biliary cholangitis (PBC). Studies suggest that a quarter of the patients suffering from limited cutaneous systemic sclerosis are positive for PBC-specific anti-mitochondrial antibodies [6]. This rare association of PBC with systemic sclerosis is often regarded as Reynold's syndrome [7]. We present an association of primary biliary cholangitis with superimposed CREST syndrome manifestations.

## Case presentation

A 40-year-old female with a past one-year history of hepatitis C presented to our medicine outpatient department (OPD) with the chief complaints of the progressive thickness and tightness of the skin of the hands and face and dysphagia for the past three months, along with arthralgia of hands for the past two months. The thickness of the skin of her hands was associated with bluish discoloration of her fingers, which worsened in cold weather and was relieved by sun exposure or wearing gloves. The dysphagia, initially for solids, later progressed for liquids as well and was associated with a sensation of food being stuck in her throat, heartburn, nausea, and vomiting. Arthralgia in the hands had persisted for the past three years and worsened for the past three months to the extent that the patient was unable to perform her regular house chores. Our patient also complained of recurrent ulcers on her fingertips, measuring 1-2 mm and associated with pain. Additional medical history includes untreated hemorrhoids and an irregular menstrual flow.

On physical examination, blood pressure was 90/70 mmHg. The face had some pigmented areas, with the nose pinched up and tapered, and there was puckering of skin around the lips with a small orifice of the mouth causing difficulty in opening the mouth. The skin of both hands was shiny, tight, thick, and edematous with pigmented and hypopigmented areas; there was decreased movement at the metacarpophalangeal and interphalangeal joints of hands. We have tabulated the remarkable laboratory findings in Table 1. 

**Table 1:** Remarkable lab findings MCV - Mean Corpuscular Volume; MCH - Mean Corpuscular Hemoglobin; WBC - White Blood Cells; IgE: Immunoglobulin E

Investigations	Results (Normal Values)
Hemoglobin	9.6 g/dl (12.0 - 16.0 g/dl)
MCV	67.3 fL (80 - 100 fL)
MCH	23 pg/cell (25.4 - 34.6 pg/cell)
WBC	11,400/mm^3^ (4500 - 11,000/mm^3^)
Lymphocytes	10% (25 - 33%)
Neutrophils	80% (54 - 62%)
IgE	2212 IU/mL (0 - 380 IU/mL)
Serum Creatinine	0.45 mg/dL (0.6 - 1.2 mg/dL)
Anti-HCV positive	59.02 IU/L (Upto 40 IU/L)
Total Serum Bilirubin	0.18 mg/dL (0.1 - 1.0 mg/dL)
Indirect Bilirubin	0.15 mg/dL (0.2 - 0.8 mg/dL)

**Figure 1:**
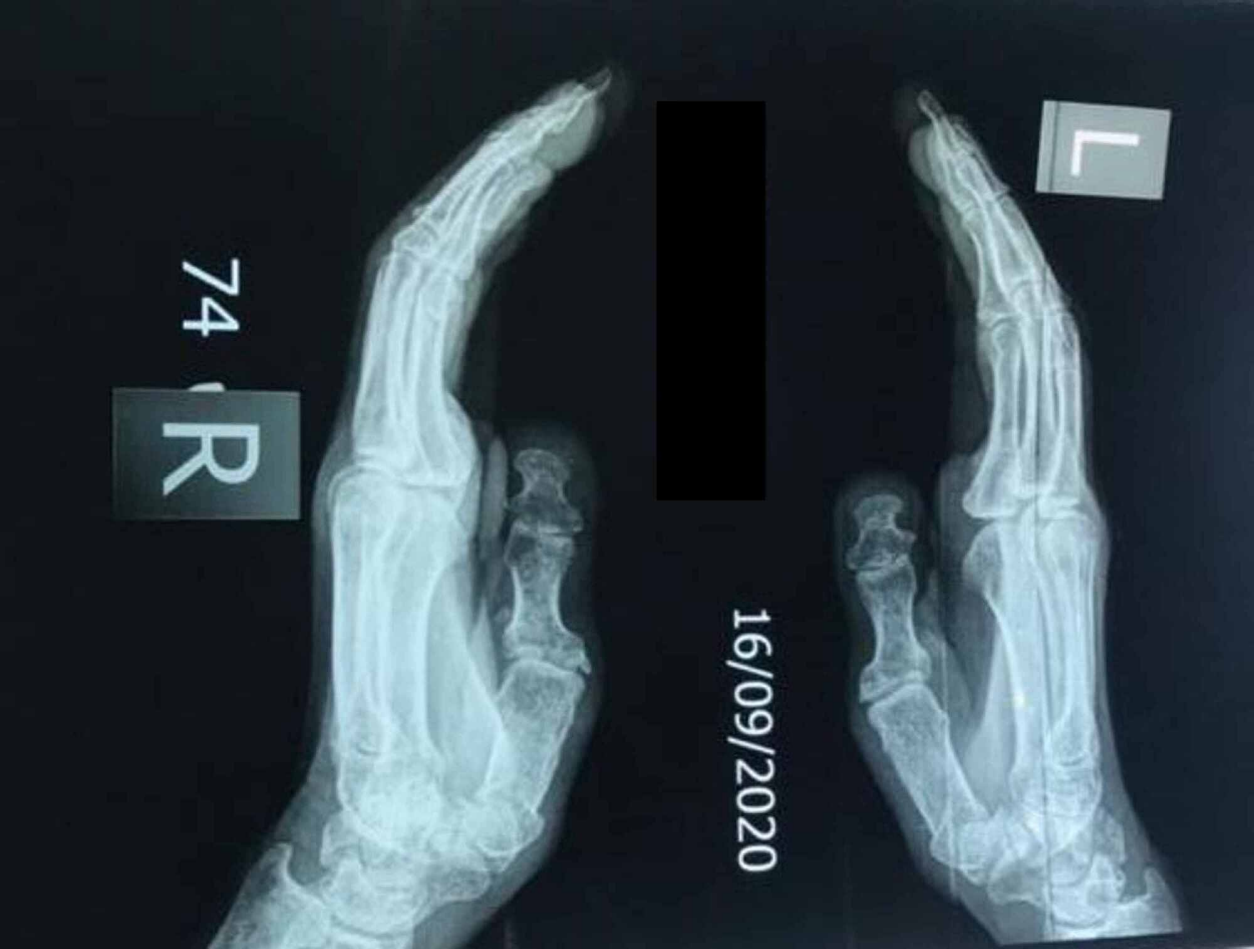
Mild osteopenia

**Figure 2:**
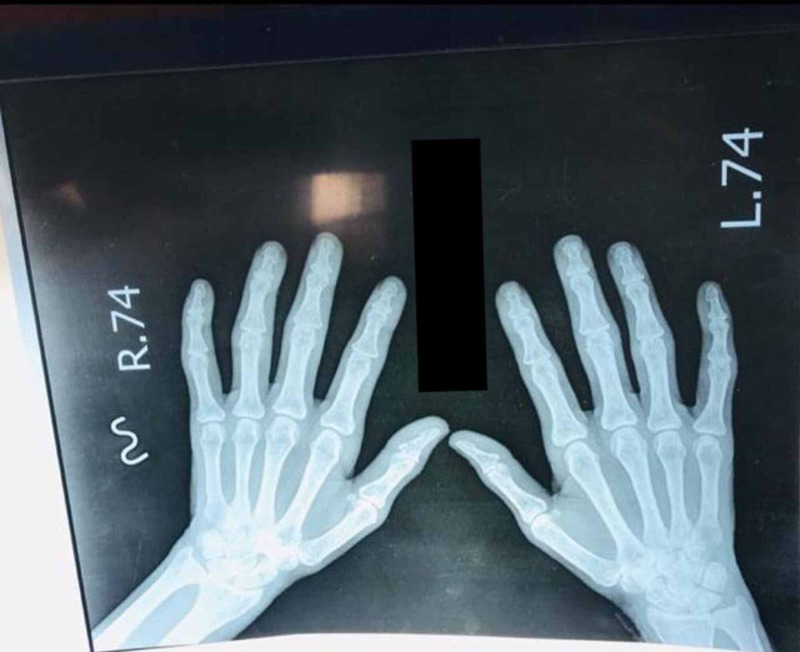
Mild osteopenia

Mild osteopenia of both hands could be appreciated on X-ray (Figures 1-2). 

**Figure 3:**
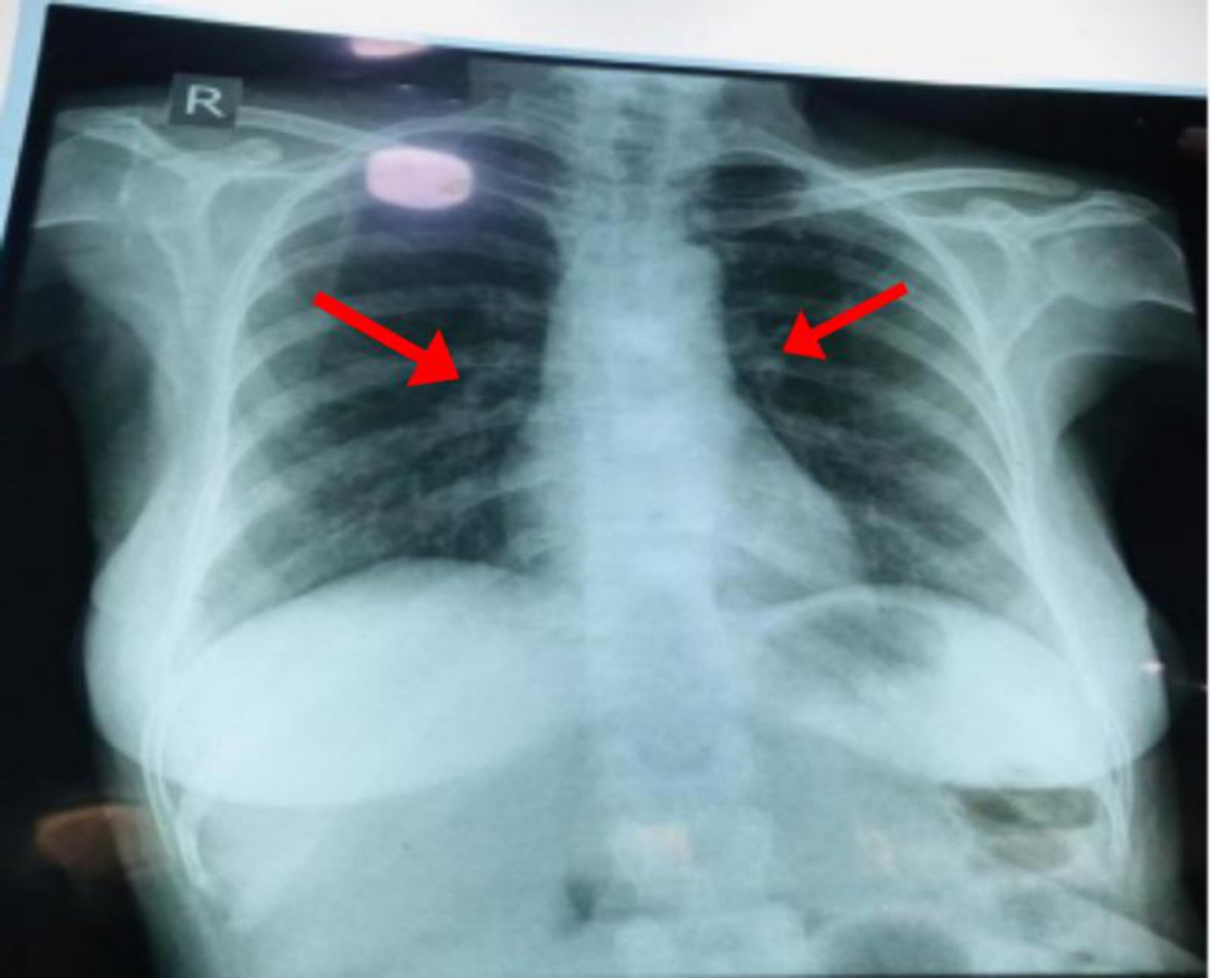
Prominent vascular markings (Arrows)

The chest X-ray (Figure 3) demonstrated prominent vascular markings probably due to pulmonary hypertension. 

On ANA profile, anti-ds DNA, anti-sm antibodies, anti-SCL 70, anti-Histone, and anti-nucleosome antibodies were all negative, and anti-CCP was < 1.50 µ/ml (<20 µ/ml). Although the patient tested positive for anti-mitochondrial M2 antibody (AMA-M2), anti centromere antibody could not be performed due to insufficiency of funds.

The patient was started on tablet omeprazole 40 mg OD, tablet piroxicam-beta-cyclodextrin BD, tablet amlodipine 5 mg OD, and tablet vitamin B complex OD. Previously, the patient had taken pegylated interferon once weekly for three months for hepatitis C.

## Discussion

Systemic sclerosis is a rare, heterogeneous, and often detrimental disorder primarily targeting the skin and may involve internal organs. With peak onset at the age of 40 years and an unusual disease in natives of Asian descent, it has a fluctuating incidence between 2.3 and 10 per million population possibly influenced by multiple factors, including environmental factors, ultimately leading to disruption of the immune mechanism along with vascular changes [1]. It was further classified by an international panel of experts in 1988 based on the location of sclerosed skin on physical examination into diffuse cutaneous systemic sclerosis (dcSSc) and limited cutaneous systemic sclerosis (lcSSc), which includes the CREST syndrome [4]. Calcinosis occurs due to the deposition of calcium hydroxy appetite aggregates chiefly involving the subcutaneous area of the finger pads and extensor surface of the elbow, which may lead to skin erosion followed by secondary infections. Raynaud's phenomena are observed in more than 95% of the patients and eventuate in response to vasospasm induced by cold. Consequent complications, such as vasculopathy, digital ulcers, and ischemia, followed by gangrene leading to autoamputation may ensue. Esophageal dysmotility noticed in 90% of the affected individuals is due to fibrosis. Hence, patients present with complaints of dyspepsia, dysphagia, and regurgitation due to the weakening of the lower esophageal sphincter.

Sclerodactyly refers to the thickening of the skin of the digits of the hands and feet. The fibrosis and thickening of the skin eventually lead to contracture, thereby reducing the mobility of the peripheral joints. Additionally, a small oral aperture is seen and is often referred to as a fish mouth or masked facies in case of facial involvement. A reliable method of identifying the extent and extremity of the thickness is the traditional modified Rodnan skin score. Telangiectasias is the dilatation of capillaries commonly appearing on the face, hands, and mucosal surface, often blanchable, and a high-risk factor for pulmonary hypertension [5]. Systemic sclerosis can be diagnosed clinically by the existence of at least three of five features of the CREST syndrome [4]. Our patient was also diagnosed clinically on the presence of Raynaud's phenomena, esophageal dysmotility, and sclerodactyly.

Literature review suggests a significant association of primary biliary cholangitis (PBC) in patients with lcSSc, with a prevalence of 2%-2.5%. Although the exact mechanism remains ambiguous, genetic, environmental, and infectious agents may be the culprits for disease progression [7]. This coexistence was initially presented in 1970 with two cases of PBC and limited scleroderma and further reinforced by Reynolds et al., who reported six cases with a similar alliance. Autoimmune association on the grounds of positive AMA antibodies in lcSSc patients was also established. Moreover, such patients require constant supervision to warrant the early diagnosis for prompt treatment [6]. Autoantibodies demonstrate a pivotal role to determine and envisage the outcome of the disease.

More than 90% of the patients with systemic sclerosis have positive ANA. Anti-centromere antibodies are more specific for diagnosing lcSSc, but due to lack of funds, this test could not be performed for our patient. ANA and AMA were positive in our patient, along with strong clinical evidence of lcSSc, thereby concluding the final diagnosis of lcSSc associated with PBC.

After a thorough review of the literature regarding the hepatological pathologies occurring in patients with systemic sclerosis, their duration with respect to systemic sclerosis, and outcomes, we have tabulated our findings in Table 2.

**Table 2:** The literature review M - Male; F - Female; UNK - Unknown

Author	Year	Age (Y)	Gender	Hepatic Pathology	Duration	Outcome
I. Marie et al. [8]	2001	67	F	Autoimmune hepatitis	After 7 years	Cessation of hepatological manifestations post-treatment
I. Marie et al. [8]	2001	48	F	Autoimmune hepatitis	Concurrent	Cessation of hepatological manifestations post-treatment
Poggi G et al. [9]	2009	55	F	Chronic hepatitis C virus infection	5 years earlier	Uneventful. Stabilization of Raynaud's phenomenon and no worsening of lung function
Simoes M et al. [10]	2011	76	M	Primary Biliary Cholangitis	After 7 years	Mortality
de Oliveira FL et al. [11]	2012	47	M	Chronic hepatitis C virus infection	2 years earlier	Stabilization of cutaneous lesions of morphea
Kiyani A et al. [12]	2017	56	F	Primary Biliary Cholangitis	Concurrent	UNK

## Conclusions

Through our case, we not only suggest the possible association of PBC with extrahepatic autoimmune disorders, such as limited cutaneous systemic sclerosis (CREST syndrome), but also hope that our case helps in the management of patients with CREST who present with superimposed hepatitis C virus infection. Such autoimmune disorders should be frequently screened, monitored, and followed up on to prevent further morbidity.
